# Evaluation of β-catenin Expression by Immunohistochemistry in Colorectal Adenoma and Carcinoma and its Correlation with Pathological Prognostic Factors

**DOI:** 10.22034/ijp.2026.2075093.3622

**Published:** 2026-08-01

**Authors:** Amrutha Shankar B, Clement Wilfred D, Soujanya H S

**Affiliations:** 1 Department of Pathology, Ramaiah Medical College, Ramaiah University of Applied Sciences, Bangalore, India

**Keywords:** Colorectal carcinoma, colorectal adenoma, prognostic factors, β-catenin.

## Abstract

**Background & Objective:**

Colorectal carcinoma (CRC) is a significant contributor to global morbidity and mortality. Identifying molecular markers for predicting CRC outcomes is crucial. β-Catenin plays a key role in the Wnt signaling pathway and acts as an essential membrane protein linked with E-cadherin, vital for cell-cell adhesion and sustaining cell polarity. This study investigated the immunohistochemical expression of β-catenin in colorectal adenoma and CRC, and its relationship with pathological prognostic indicators.

**Methods:**

This cross-sectional study analyzed 55 adenoma and 45 CRC cases. Tumor morphology was examined using light microscopy. Chromogenic immunohistochemistry (IHC) technique was used to evaluate β-catenin expression.

**Results:**

Among 55 adenoma and 45 CRC cases, a significant link was found between β-catenin expression and tumor categorization. Malignant tumors exhibited higher nuclear β-catenin levels. Reduced membrane β-catenin expression was associated with deeper tumor invasion. A notable association was observed between nuclear β-catenin patterns and lymph node involvement, with higher nuclear levels indicating an increased likelihood of lymph node metastasis. Additionally, the study highlighted the relationship between β-catenin expression and TNM staging: early-stage disease showed higher membrane-bound β-catenin, while advanced stages had increased nuclear β-catenin.

**Conclusion:**

This study demonstrated a shift in β-catenin expression from the membrane in normal tissues to the nucleus in adenomas and CRCs. This progression underscores β-catenin’s role in evaluating colorectal cancer malignancy risk, supporting early intervention. The gradational increase in nuclear β-catenin showed an association with advancing cancer stages, establishing it as a potential prognostic marker.

## Introduction

Colorectal carcinoma (CRC) is a significant global health concern, contributing substantially to morbidity and mortality, with approximately 1.2 million new cases and 600,000 deaths reported annually(1). It is the second leading cause of cancer-related mortality worldwide, with nearly 50% of patients dying due to complications related to tumor invasion and metastasis(2). In India, CRC ranks as the fourth most common cancer in males and the third most common in females(1). Early detection plays a crucial role in reducing mortality and improving survival rates, thereby highlighting the need for increased research into reliable diagnostic markers(1–3). Despite considerable advances in diagnostic and therapeutic approaches, the prognosis of advanced CRC remains poor(2,3). Although several clinicopathological parameters, such as the extent of local tumor spread and lymph node involvement, are well-established prognostic factors, identifying patients at high risk of postoperative recurrence continues to be challenging(3). Therefore, there is a need to identify molecular markers that can serve as effective predictive and prognostic indicators in CRC.

β-Catenin is an important component of the Wnt signaling pathway(2,4). It is a membrane-associated protein linked to E-cadherin and plays a vital role in maintaining cell-cell adhesion and cellular polarity(1–4). Alterations or mutations in components of the Wnt pathway lead to cytoplasmic accumulation and subsequent nuclear translocation of β-catenin. In the nucleus, it functions as a transcriptional activator, promoting the expression of genes involved in tumor invasion and progression, thereby acting as a key oncoprotein in colorectal carcinogenesis(2,5). The intracellular localization of β-catenin significantly influences tumor behavior and phenotype; loss of membranous expression along with increased nuclear accumulation is associated with enhanced proliferative and invasive potential(2,5). Studies conducted internationally have demonstrated that increased nuclear expression and reduced membranous expression of β-catenin are associated with aggressive tumor characteristics, higher rates of lymph node metastasis, decreased overall survival, and progression to advanced stages of CRC(2,4–6). In addition to genetic factors, lifestyle influences—particularly diet—play an important role in colorectal cancer progression. Recent evidence suggests that dietary patterns can modulate tumor biology and the microenvironment. These external factors may interact with oncogenic pathways such as Wnt/β-catenin signaling, which is central to carcinogenesis and metastasis. Dietary acid load, reflecting long-term nutritional exposure, may contribute to metabolic and inflammatory changes that influence β-catenin activity and disease progression(7).

However, further research is required to establish the role of β-catenin as a reliable diagnostic marker in CRC. Moreover, due to the limited number of studies conducted in the Indian population, the present study aims to evaluate the immunohistochemical expression and diagnostic significance of the differential intracellular localization of β-catenin in colorectal neoplasms.

## Materials and Methods

The data for the cross-sectional observational study were obtained from patients aged >18 years with radical resection specimens of carcinomas, including segmental colectomy, hemicolectomy, anterior resection and abdominoperineal resection, and polypectomy specimens of adenomas were included. Biopsy, endoscopic mucosal resection, or polypectomy specimens for carcinomas were excluded due to incomplete prognostic assessment. Cases with extensive tumor necrosis and insufficient viable tumor cells for immunohistochemical evaluation were also excluded. The data collection period spanned from August 2022 to July 2024. The study was conducted after obtaining approval from the Institutional Ethics Committee, and informed consent was obtained from all participants.

### Method of Data Collection

The polypectomy specimens and resection specimens of CRCs were received in the Pathology Department in 10% formalin. In every case, the standard protocol for surgical grossing of resected specimens was followed. For CRCs, after meticulous gross examination, multiple representative tissue bits were taken from the tumor, surgical margins, mesocolon, and all lymph nodes. Then the tissue bits were processed as per standard protocol, and 5-µm-thick sections were prepared from the formalin-fixed paraffin-embedded blocks and stained with hematoxylin and eosin (H&E) stain. The H&E-stained slides were studied for tumor histology, grade, lymphovascular invasion, lymph node metastasis, and other histologic features and were staged according to the American Joint Committee on Cancer staging system (8th edition, 2017). For colorectal adenomas, the H&E-stained slides were evaluated for histologic type and degree of dysplasia.

### Processing for Immunohistochemistry (IHC)

Immunohistochemical staining for β-catenin was performed on formalin-fixed, paraffin-embedded tissue sections of 4-µm thickness placed on coated glass slides. The sections were deparaffinized in xylene for three changes of 10 minutes each and rehydrated through graded alcohols of 100%, 95%, 80%, 70%, and 50%, with each step carried out for 10 minutes, followed by rinsing in buffer. Antigen retrieval was carried out using Tris-EDTA buffer in a microwave oven using repeated heating and cooling cycles. Endogenous peroxidase activity was blocked using 3% hydrogen peroxide for 15 minutes, followed by application of a protein block for 15 minutes. The sections were then incubated with a pretitrated primary monoclonal antibody against β-catenin, clone EP35, obtained from PathnSitu, for 45–60 minutes. Subsequently, the sections were treated with a post-primary block for 30 minutes and polymer-based secondary antibody, typically poly-HRP. Visualization was achieved using diaminobenzidine as the chromogen for 5 minutes. The slides were counterstained with hematoxylin for 20 seconds, mounted with DPX, and preserved for microscopic examination. Appropriate controls were included with each batch, and all incubation steps were carried out in a moist chamber with intermittent washes in Tris-buffered saline to ensure optimal staining and antigen visualization.

The expression of β-catenin was assessed by comparing staining patterns in normal mucosa and neoplastic tissue. A positive immunoreaction was identified by brown membranous staining in histologically normal colorectal tissue, serving as the internal control, and dark brown to black nuclear staining in fibroblasts of desmoid tumors, which acted as the external control. Immunohistochemistry (IHC) was performed on whole tissue sections from 100 cases. Evaluation of staining was conducted in four microscopic fields, encompassing approximately 1000 cells, using a light microscope at 400× magnification. All slides were independently evaluated by two experienced pathologists blinded to clinical data. Expression patterns were categorized as membranous, cytoplasmic, and nuclear. The scoring of β-catenin expression was performed according to staining intensity, categorized as 0 (no expression), 1+ (weak), 2+ (moderate), 3+ (strong), and 4+ (very strong). The final consensus IHC score, ranging from 0 to 400, was determined by multiplying the percentage of positively stained cells or nuclei by the corresponding staining intensity(1).

### Statistical Analysis of Data

Data were analyzed using SPSS version 22. Categorical variables were expressed as frequencies and proportions and analyzed using the χ² test or Fisher exact test for 2 × 2 tables. Continuous variables were expressed as mean ± standard deviation. Independent t test and ANOVA were used for comparisons. A P value <0.05 was considered statistically significant.

## Results

This study comprised 55 cases of adenoma and 45 cases of carcinoma. The mean age of patients was 53.8 years, with the majority of cases occurring in the sixth decade. A statistically significant difference, as assessed by independent t test, was observed between genders across different age groups, indicating a higher distribution of colorectal adenomas in older men and younger women. Furthermore, the distribution of colorectal adenomas was greater in men than in women, with a male-to-female ratio of 2.43. Of 55 adenoma cases, low-grade dysplasia accounted for 80% of cases, while high-grade dysplasia comprised 20%. In the adenoma group, the tubular histological pattern was predominant, representing 78.2% of cases.

The average age of carcinoma patients was 55.53 years, with the highest distribution (28.9%) observed in individuals in their seventies. Furthermore, colon carcinoma was more common in males than in females, with a ratio of 1.6:1. Other clinicopathological features of the carcinoma cases are depicted in Table 1. The differences observed between low- and high-grade adenomas in β-catenin expression scores were not statistically significant. The analysis, which focused on histological patterns in colorectal adenomas and their expression of β-catenin, revealed no statistically significant differences.

**Table 1:** Histopathological features of colorectal carcinoma

Features		Frequency (Percentage)
Gross appearance	Non–proliferative	28 (62.2%)
	Proliferative	17 (37.8%)
Histological Type	Adenocarcinoma NOS	38 (84.4%)
	Mucinous Adenocarcinoma	5 (11.1%)
	Signet ring cell carcinoma	2 (4.4%)
pT stage	pT2	9 (20.0%)
	pT3	26 (57.8 %)
	pT4	10 (22.2%)
Histological grade	Grade I	10 (22.2%)
	Grade II	28 (62.2%)
	Grade III	7 (15.6%)
Lymph node status	Positive	18 (40.0%)
	Negative	27 (60.0%)
TNM Stage	Stage I	5 (11.1%)
	Stage II	20 (44.4%)
	Stage III	18 (40.0%)
	Stage IV	2 (4.4%)

**Fig. 1:**
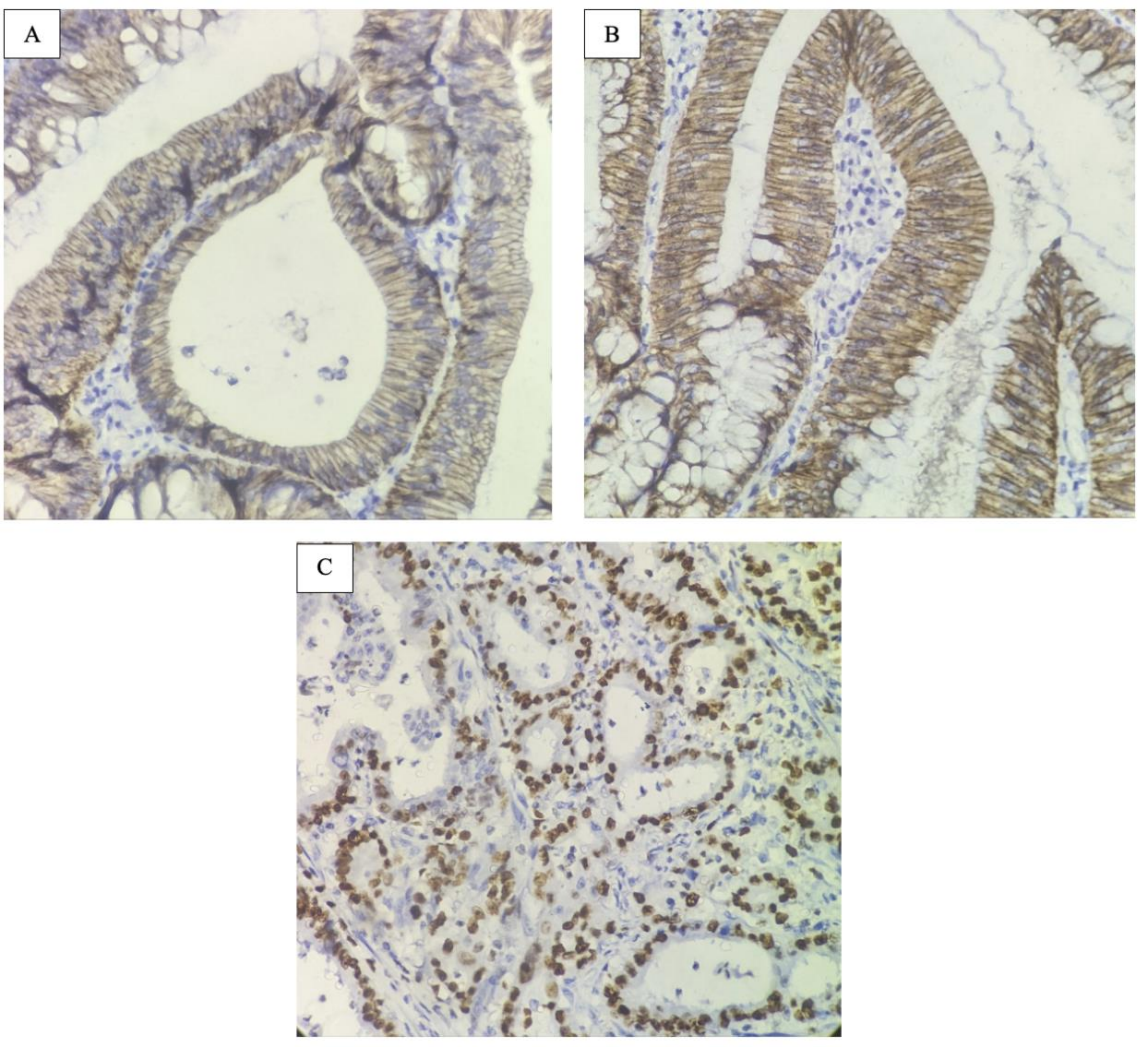
A) Membranous expression of β-catenin in low-grade tubular adenoma (IHC, 40×). B) Cytoplasmic localization of β-catenin in low-grade villous adenoma (IHC, 40×). C) Nuclear localization of β-catenin in adenocarcinoma (IHC, 40×).

The study found a statistically significant association between the cellular localization and scored intensity of β-catenin expression and the classification of colorectal tumors into benign or malignant categories (P < 0.001) (Table 2). Malignant tumors showed a higher average score for nuclear β-catenin expression. Additionally, there was a clear increase in the β-catenin nuclear score within malignant tumors, demonstrating a progression from membranous localization to cytoplasmic and finally to nuclear positivity (Fig. 1).

**Table 2:** Comparison of β-catenin expression between colorectal adenoma and carcinoma groups

β-Catenin Score	Colorectal Neoplasms	Mean	±SD	p-value (Independent t test)
Membranous score	Benign	153.18	15.763	<0.001
	Malignant	26.00	18.998	
Cytoplasmic score	Benign	106.09	19.310	0.002
	Malignant	120.89	26.953	
Nuclear score	Benign	22.64	9.372	<0.001
	Malignant	205.78	34.607	

No statistically significant relationship was found between β-catenin expression and age or sex in carcinoma cases. Similarly, no statistically significant association was identified with gross appearance, tumor size, histological type, tumor grade, or perineural invasion. The study identified a statistically significant association between membranous expression of β-catenin and the depth of tumor invasion, showing that an increase in tumor depth led to a decrease in the membranous score (Table 3). However, the mean membranous score for pT2 cases was 46, with a relatively large standard deviation (SD = 33), indicating heterogeneity in membranous β-catenin expression within this subgroup.

**Table 3:** Expression of β-catenin in colorectal carcinoma with respect to depth of invasion

Depth of invasion	Membranous Score		Cytoplasmic Score		Nuclear score	
	Mean	±SD	Mean	±SD	Mean	±SD
pT2	46	33	127	32	189	46
pT3	21	9	119	27	211	32
pT4	21	9	120	25	209	28
p-value (ANOVA test)	0.001		0.778		0.264	

SD, standard deviation. Scores range 0–400 (percentage of positive cells × intensity).

There was a statistically significant relationship between membranous and nuclear β-catenin expression and lymph node status, with a P value <0.001 (Table 4). This finding indicated that higher membranous scores were associated with the absence of lymph node involvement, whereas increased nuclear scores were linked to the presence of lymph node metastasis. The study also demonstrated a statistically significant association between β-catenin expression, both membranous and nuclear, and TNM stage classification (P < 0.001) (Table 5), showing that early stages (I and II) exhibited higher membranous scores, while advanced stages (III and IV) showed increased nuclear scores.

**Table 4:** Expression of β-catenin in colorectal carcinoma with respect to lymph node status

Lymph Node Status	Membranous Score		Cytoplasmic Score		Nuclear score	
	Mean	±SD	Mean	±SD	Mean	±SD
Negative	34	21	124	20	191	30
Positive	14	3	116	35	228	29
p-value (ANOVA test)	<0.001		0.284		<0.001	

**Table 5:** Expression of β-catenin in colorectal carcinoma with respect to TNM Stage

STAGE GROUPING	Membranous Score		Cytoplasmic Score		Nuclear score	
	Mean	±SD	Mean	±SD	Mean	±SD
Stage I	67	29	146	26	160	28
Stage II	28	7	120	12	191	11
Stage III	14	3	114	36	230	31
Stage IV	10	0	125	35	255	21
p-value (ANOVA test)	<0.001		0.140		<0.001	

## Discussion

β-Catenin, a key component of the Wnt signaling pathway, plays an essential role in cell-cell adhesion and maintenance of cellular polarity(1–4). Dysregulation of this pathway results in cytoplasmic accumulation and nuclear translocation of β-catenin, promoting oncogenic gene expression and colorectal carcinogenesis(2–5). Loss of membranous localization further enhances tumor invasiveness and proliferation(2,5). The present study evaluated β-catenin expression in relation to pathological prognostic factors in colorectal neoplasms.

In the present study, colorectal adenomas showed a male predominance, with a higher male-to-female ratio compared to findings by Valian et al. and Wernly et al.(8,9). The mean age was comparable to studies by Bhattacharya et al. and Khairy et al.(1,10). A significant gender-based difference in age distribution was observed (P = 0.024), which contrasts with findings reported by Lee et al.(11), highlighting variability in demographic patterns. Low-grade dysplasia was predominant (80%), exceeding proportions reported by Bhattacharya et al., Khairy et al., and Zhan et al.(1,10,12), suggesting differences in case distribution.

Tubular adenoma was the most common histological subtype, differing from Khairy et al., who reported tubulovillous adenoma as the predominant type(10). Adenomas were most frequently located in the rectum and sigmoid colon, whereas Khairy et al. reported a higher prevalence in the left colon(10), indicating variability in anatomical distribution.

The mean age of colorectal carcinoma (CRC) patients was 55.53 years. The proportion of younger patients was lower than reported by Magadhi et al.(13).

However, recent studies(14) suggest an increasing incidence of CRC in younger individuals, possibly due to lifestyle and environmental factors. The mean age of CRC was comparable to Hajmanoochehri et al. and Gullickson et al.(15,16), but higher than Magadhi et al. and lower than Khairy et al.(13,10). A male predominance was observed, consistent with Magadhi et al., but contrasting with Khairy et al.(13,10). These variations may reflect geographical and lifestyle differences(17- 19).

Most CRC cases were located in the rectosigmoid region, consistent with findings by Magadhi et al.(13), whereas Khairy et al. reported a predominance in the right colon(10). Conventional adenocarcinoma was the most common histological type, in agreement with Bhattacharya et al., Khairy et al., Magadhi et al., and Gullickson et al.(1,10,13,15). Mucinous and signet ring cell carcinomas were less frequent but are known to be associated with poorer prognosis(20–22).

Tumor invasion depth is an important prognostic factor, with increasing depth associated with higher risk of vascular and lymphatic spread(23). In this study, pT3 tumors were predominant, consistent with findings by Khairy et al., and Magadhi et al.(10,13). Lymph node metastasis was observed in 40% of cases, comparable to Bourroul et al. and Khairy et al., but lower than Magadhi et al. and Wong et al.(24,25,10,13,4). Most cases were classified as stage II and III, consistent with Yoshida et al., Wong et al., and Khairy et al.(3,4,10).

A significant association was observed between β-catenin expression and tumor classification, benign versus malignant (P < 0.001), with malignant tumors demonstrating increased nuclear expression. A progressive shift from membranous to cytoplasmic and nuclear localization was noted, consistent with findings by Bhattacharya et al. and Panda et al.(1,26).

In adenomas, β-catenin expression did not show significant variation, in agreement with Bhattacharya et al.(1). However, Panda et al. reported reduced membranous expression and increased cytoplasmic localization in higher grades of dysplasia(26). Differences may be attributed to variations in sample size and dysplasia grade distribution. Furthermore, Guney et al. and Garalla et al. reported variability in β-catenin expression across adenoma subtypes(27,28), highlighting tumor heterogeneity.

No significant association was found between β-catenin expression and age, gender, tumor size, or histological type, consistent with findings by Bhattacharya et al., Gao et al., and Guney et al.(1,2,27). However, Chiang et al. and Peker et al. reported differing results(29,30), suggesting variability across studies. Similarly, no significant association was observed with tumor grade, although Guney et al., Garalla et al., and Peker et al. reported increased nuclear expression in poorly differentiated tumors(27,28,30).

A significant association was observed between decreased membranous β-catenin expression and increasing tumor invasion depth, indicating its role in tumor aggressiveness. This finding contrasts with Gao et al. and Chiang et al.(2,28). Additionally, a significant correlation was found between β-catenin expression and lymph node status (P < 0.001), with increased nuclear expression associated with nodal metastasis. These findings are consistent with Wong et al., Guney et al., and Peker et al., although Panda et al. and Mukherjee et al. reported differing results(4,26,27,30-32).

No significant association was observed with perineural invasion, consistent with Panda et al.(26). However, a significant correlation was found between β-catenin expression and TNM staging (P < 0.001). Early-stage tumors predominantly exhibited membranous expression, whereas advanced-stage tumors showed increased nuclear localization. Similar findings were reported by Bhattacharya et al., Gao et al., and Garalla et al.(1,2,28), although Panda et al. reported contrasting results(26). These findings may aid in risk stratification and guide targeted therapeutic strategies in colorectal carcinoma.

. However, variability in expression patterns across studies suggests the influence of additional factors. Emerging evidence indicates that environmental and lifestyle factors, particularly dietary patterns, may modulate key molecular pathways involved in carcinogenesis(7). These interactions may contribute to population-based differences in tumor behavior.

Our findings further support the pivotal role of aberrant Wnt/β-catenin signaling in colorectal tumorigenesis. The progressive increase in β-catenin expression from adenoma to carcinoma observed in this study is consistent with evidence that Wnt pathway dysregulation is an early event in colorectal carcinogenesis. Recent advances have highlighted the potential of β-catenin and other Wnt pathway components as diagnostic, prognostic, and therapeutic biomarkers. Although our study did not assess clinical outcomes, the observed alterations in β-catenin expression support its potential as a biomarker in colorectal neoplasia(33). Although the underlying mechanisms were not explored in the present study, further research is warranted to elucidate the interplay between molecular alterations and environmental influences in colorectal cancer.

## Conclusion

The study documented a steady change in the subcellular location of β-catenin expression from colorectal adenomas to carcinomas. In adenomas, β-catenin was primarily found in the membrane and cytoplasm, a difference that was statistically significant. Conversely, in carcinomas, there was a greater presence of β-catenin in the nucleus. This pattern suggests that the movement of β-catenin from the membrane to the nucleus is linked to enhanced malignant potential and progression in the adenoma-carcinoma sequence. The research identified a statistically significant relationship between the decrease in the membranous score of β-catenin expression and the increase in tumor invasion depth, indicating that the membranous score lowers as tumor depth increases. Carcinoma cases without lymph node metastasis exhibited higher membranous scores, while those with lymph node metastasis showed higher nuclear scores, indicating that increased nuclear staining is linked to the presence of nodal metastasis. Furthermore, the significant and positive relationship between increased nuclear scores of β-catenin in advanced stages (AJCC-TNM), which is the key prognostic indicator for colon cancer, advocates for the inclusion of β-catenin immunohistochemistry as a supplementary prognostic tool in colorectal adenocarcinoma. This supports the notion that evaluating the nuclear localization of β-catenin using immunohistochemical methods can provide important prognostic information, complement existing staging techniques and potentially guide more tailored treatment approaches for patients with this type of cancer. Therefore, these results indicate that β-catenin presents a promising therapeutic target. By focusing on this target, it could lead to the optimal treatment of both cancerous and precancerous cells, while minimizing harm to the nearby healthy mucosa. Nonetheless, to definitively confirm β-catenin’s role as both a therapeutic and prognostic marker in colorectal cancer, additional research involving larger populations and survival analyses is required.

## Data Availability

The data that support the findings of this study are available from the corresponding author upon request.
